# High‐throughput whole‐slide scanning to enable large‐scale data repository building

**DOI:** 10.1002/path.5923

**Published:** 2022-06-08

**Authors:** Mark D Zarella, Keysabelis Rivera Alvarez

**Affiliations:** ^1^ Department of Pathology Johns Hopkins University Baltimore MD USA; ^2^ Department of Biomedical Engineering Johns Hopkins University Baltimore MD USA

**Keywords:** digital pathology, virtual slide, whole‐slide imaging, whole‐slide scanning, artificial intelligence, machine learning, computational pathology, data repository

## Abstract

Digital pathology and artificial intelligence (AI) rely on digitization of patient material as a necessary first step. AI development benefits from large sample sizes and diverse cohorts, and therefore efforts to digitize glass slides must meet these needs in an efficient and cost‐effective manner. Technical innovation in whole‐slide imaging has enabled high‐throughput slide scanning through the coordinated increase in scanner capacity, speed, and automation. Combining these hardware innovations with automated informatics approaches has enabled more efficient workflows and the opportunity to provide higher‐quality imaging data using fewer personnel. Here we review several practical considerations for deploying high‐throughput scanning and we present strategies to increase efficiency with a focus on quality. Finally, we review remaining challenges and issue a call to vendors to innovate in the areas of automation and quality control in order to make high‐throughput scanning realizable to laboratories with limited resources. © 2022 The Authors. *The Journal of Pathology* published by John Wiley & Sons Ltd on behalf of The Pathological Society of Great Britain and Ireland.

## Introduction

Whole‐slide imaging (WSI) is a method used to digitize glass slides, producing digital images of histology that are commonly used to support education, collaboration, digital archiving, and research [1]. WSI has also been featured in the clinical workflow to support activities such as telepathology [2, 3, 4, 5, 6], remote signout [7, 8, 9], and quantitation [10, 11, 12]. As clinical use cases for WSI continue to emerge, it is expected that adoption of digital pathology will experience continued expansion. Likewise, as artificial intelligence and machine learning (AI/ML) activities become more ubiquitous, the need for large image repositories will also grow, requiring a high‐throughput method for scalable data sourcing.

**Figure 1:**
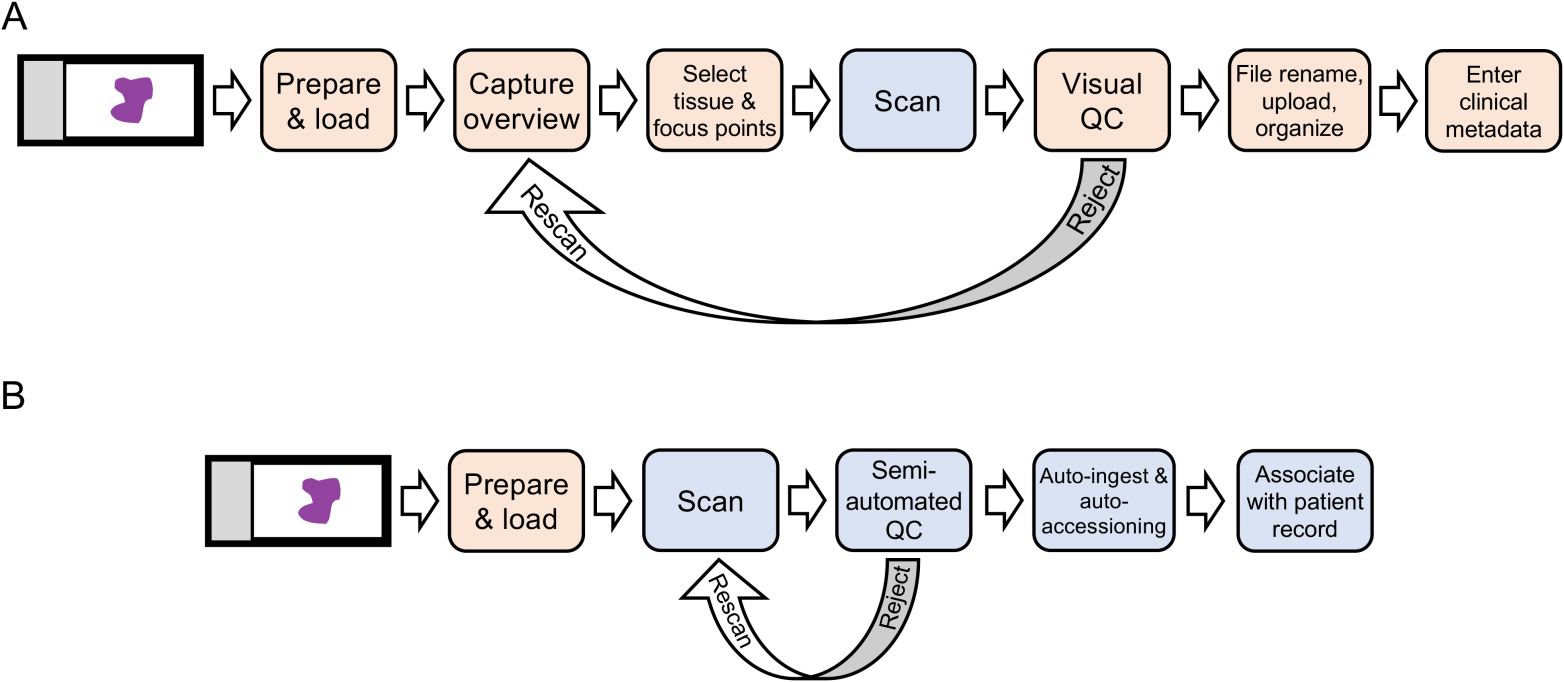
Conventional versus automated slide scanning. (A) A conventional scanning workflow involves significant pre‐ and post‐scanning interaction. (B) Automation leverages image analysis, streamlined QC, and automated processing. Steps that require manual intervention are represented in tan. Automated steps are represented in blue.

WSI has historically been an interactive process in which operators guide slide scanning by adjusting scan settings, selecting scan areas, performing pre‐scan quality verification, entering data into metadata fields, accessioning the image into a ‘case’ or other organizational structure, and performing post‐scan quality assessment. Traditionally, whole‐slide scanners are accompanied by dedicated computers that provide much of this functionality. A conventional scanning workflow is depicted in Figure 1A. After slide curation, operators prepare and load slides into the scanner and capture a slide overview image which provides them the opportunity to adjust scan parameters on a per‐slide basis in an interactive fashion. After the scan is complete, the operator usually evaluates the image produced. In order for the scanned image to be useful, it also must be organized in some fashion either via file hierarchies and naming convention, metadata tagging, or specialized image management platforms.

Together, these steps require significant manual intervention. Conventional wisdom has suggested that a laboratory needs 0.5 FTEs (full‐time equivalents) to operate each whole‐slide scanner [13], although this number greatly depends on the complexity of the workflow, the capacity of the scanner, and the automation available. A more relevant metric may be scanned slides per person‐hour, which generally should also include quality control (QC) and post‐scanning activities, even if carried out by other personnel. It must also account for rescanning slides of insufficient image quality, which not only potentially requires multiple scans per slide but may also require additional manual steps like pulling a slide from archives (depending on when the QC takes place).

In this paper, we describe innovations in WSI technology and workflows that have taken place and others that may still need to be developed by vendors and laboratories to achieve a high‐throughput workflow.

## High‐throughput whole‐slide scanning

**Figure 2:**
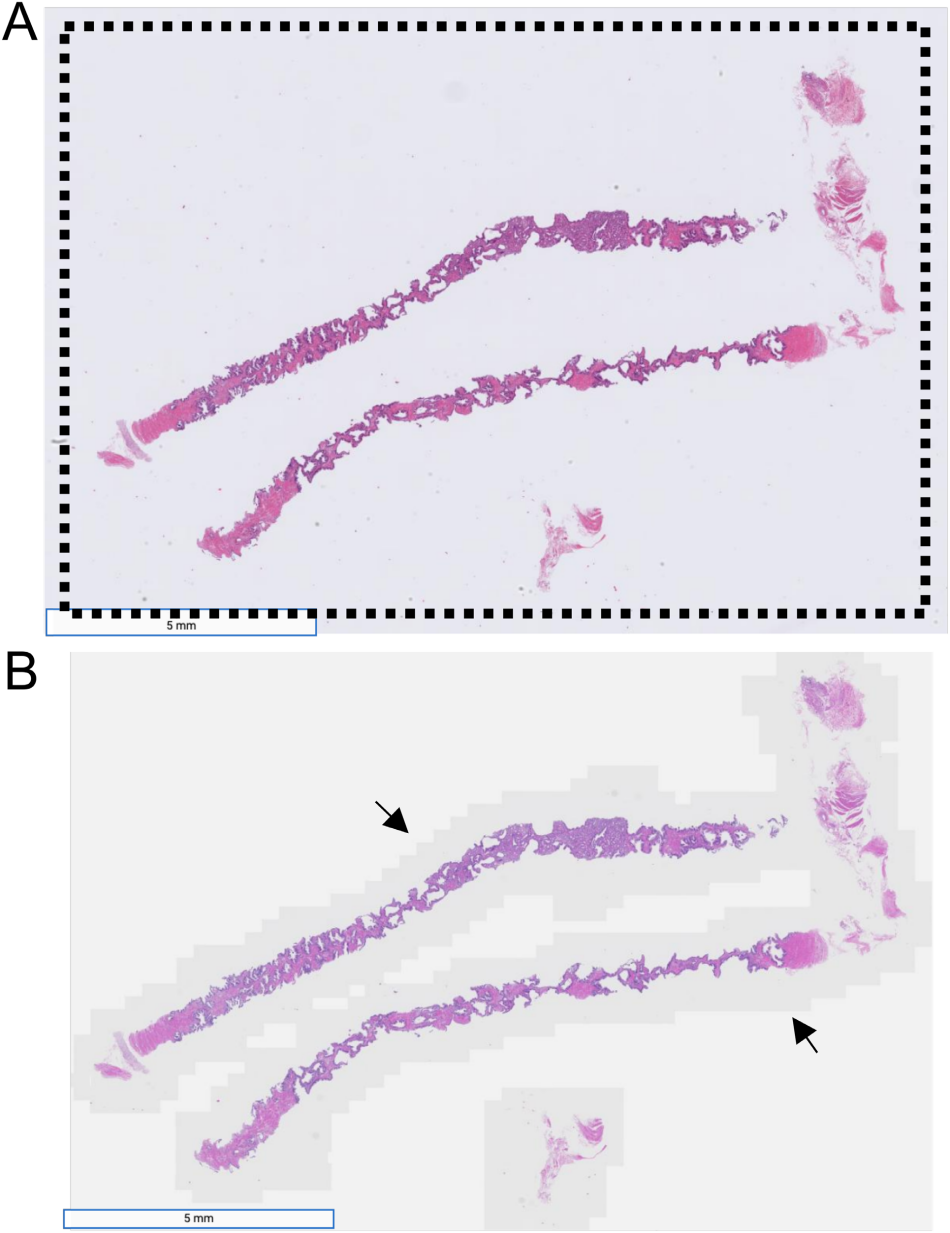
Slide scanning area is impacted by tissue geometry. (A) A line scanner scans regions of the slide within one or more bounding boxes positioned around the tissue. An example is depicted by the dotted line. Sample image was acquired using a Hamamatsu NanoZoomer S360. (B) A tile scanner captures tiles of fixed size surrounding the tissue with a padding value that can be customized in some scanners. This has the potential to reduce scan time. Regions in darker gray as indicated by the arrows depict blank regions of the slide that were scanned, whereas the lighter regions represent unscanned areas. Sample image was from the same slide as A but acquired using a Zeiss AxioScan Z1.

High‐throughput slide scanning can be distinguished from conventional slide scanning by the presence of all three of the following factors:

1. *Scan speed*: the scanner must be able to scan slides quickly at the target magnification. Although manufacturers often cite the 15 × 15 scan speed (the time it takes to scan a 15 × 15 mm square region in the slide), there are several factors that are often not captured by this metric. For example, the geometry of the tissue on the slide can impact the time it takes to scan a slide, particularly for line scanners (as opposed to tile scanners, Figure 2). This effectively makes the scan area larger, leading to longer scan times. Also, other factors such as the time it takes for the scanner to load the slide onto the stage can vary considerably across scanners, and in many cases pre‐scan snapshots, focal plane capture, and file post‐processing can be a significant factor in the duration of a scan, which may not necessarily be accounted for in the manufacturer's specification. There are a multitude of other factors as well that can impact speed considerably, such as tile overlap, objective magnification, focal plane range and precision, tissue detection sensitivity, geometric parameters, and others. Therefore, the ‘real world’ speed of a scanner on slides likely to be encountered in practice should be assessed before predicting throughput. To demonstrate how this may vary across modern scanners, we measured the scan time from nine sample slides scanned on four different scanners (Table 1). The fastest scanner that we tested usually scanned slides in under 2 min, and for some slides, under 1 min. The two slowest scanners were 7.4× slower, on average, with particularly long scan times for large tissue sections.
2. *Capacity*: the scanner capacity must be large enough to enable batch scanning without the need for frequent human interaction. Many modern whole‐slide scanners can accommodate several hundred to a thousand slides per batch. The optimal capacity for a high‐throughput operation should be built around the anticipated volume for the project. For example, if only 400 slides are loaded by staff per day, a 1,000‐slide capacity scanner will likely have a lot of unused capacity. Conversely, a single 100‐slide scanner may also not be optimal, as the staff would have to unload/reload the scanner several times per day to accommodate their volume. Depending on budget, available space, and the needs of the project, several smaller‐capacity scanners may sometimes be preferable to a single large‐capacity scanner, as they can operate in parallel, therefore scanning more quickly (all else being equal) and enabling users to mitigate downtime by scanning on the remaining scanners in case of scanner failure. It may also provide a more flexible method for prioritizing urgent scans, although many manufacturers have built such functionality into their scanners already.
3. *Automation*: a high‐throughput operation fundamentally relies on the ability to batch scan multiple slides quickly without significant user intervention or excessive setup time. This requires a degree of automation that, at minimum, relies on accurate automatic tissue detection and focusing but also benefits from other factors as described in the following section.

**Table 1:** Common whole‐slide scanner speeds and throughput

	A			B			C			D		
Scanner	Scan time (s)	Size (mm^2^)	Norm. time	Scan time (s)	Size (mm^2^)	Norm. time	Scan time (s)	Size (mm^2^)	Norm. time	Scan time (s)	Size (mm^2^)	Norm. time
Resection												
1	66	479.4	31	257	692.1	83.6	541	537.1	226.6	1,022	506.2	454.2
2	87	695.7	28.1	272	677.1	90.4	778	777	225.3	1,150	448.8	576.6
3	67	490.7	30.7	195	457.7	95.9	528	571.7	207.8	905	439.6	463.2
Biopsy												
4	33	157.9	47	94	156.8	134.9	250	184.8	304.4	302	111.7	608.2
5	42	266.5	35.5	126	353.9	80.1	384	425.2	203.2	417	100	938
6	15	67.7	49.9	76	81.3	210.4	92	84	246.4	186	67.2	622.4
IHC												
7	120	768	35.2	184	377.5	109.7	810	897.7	203	959	290.9	741.7
8	108	625.2	38.9	150	486	69.4	681	803.2	190.8	775	199.2	875.4
9	11	41.4	59.8	37	34.6	240.6	81	57.7	315.6	207	36.7	1,268.9
Capacity	360 slides			6 slides			210 slides			100 slides		

We tested four scanners on a set of nine slides: three H&E‐stained resections, three H&E‐stained biopsies, and three immunohistochemistry (IHC) slides. Slides were purposefully selected to test a range of tissue geometries, and scanners were selected to demonstrate the impact of different strategies on scan time, noting that not all of the scanners tested share the attributes required to be considered ‘high throughput’. Pixel size of the generated images ranged from 0.22 to 0.25 μm per pixel. Normalized time (norm. time) represents the estimated time it would have taken to scan a 15 × 15 mm area of tissue, calculated by dividing scan time by tissue area and multiplying by 225 mm^2^. All images were confirmed to be of sufficient quality and to have captured the tissue in its entirety. Discrepancies in image size across scanners were due mostly to different scanning strategies employed, where some scanners conformed tight to the boundaries of the tissue or discarded blank space between biopsy cores, for example. The scanners were as follows: (A) Hamamatsu NanoZoomer S360 (Hamamatsu Photonics K.K., Hamamatsu City, Japan); (B) Roche VENTANA DP200 (Ventana Medical Systems, Oro Valley, AZ, USA); (C) Hamamatsu NanoZoomer S210 (Hamamatsu Photonics K.K.); and (D) Zeiss AxioScan Z1 (Zeiss, Oberkochen, Germany).

## Automation to enable efficient workflows

### Tissue detection and focusing

As noted, automation is an essential part of an efficient high‐throughput workflow. Pivotal to scanning automation is automatic tissue detection and focusing. Accuracy is paramount, as failure at either step results in the potential to exclude tissue from the whole‐slide image or to introduce out‐of‐focus regions of the slide. In most modern scanners, reducing the likelihood of failure can be improved by increasing tissue detection sensitivity and focus precision parameters, respectively, but often at the expense of scan speed. For example, tissue detection thresholds can be made more sensitive to ensure that all tissue regions are captured, but if made too sensitive can result in large swaths of the slide scanned that do not contain tissue, which can increase scan duration considerably. Similarly, tissue detection algorithms that rely on accurate color profiling of tissue may do better at rejecting objects in the slide that are not tissue (e.g. slide labels) but may also have difficulty capturing certain types of tissue or stains.

Likewise, focus range and precision can be adjusted, number of focus points (for focus map generating scanners), and other parameters impact precision of focus, but typically at the expense of scan speed. High‐throughput scanning relies not only on selecting an appropriate balance of parameters but also on the accuracy of the scanner's algorithms to execute these functions. Many manufacturers have identified this as an area of importance and the algorithms have become more robust or even more automated (e.g. by replacing a static tissue detection threshold with automatic threshold detection), but further innovation in this area will be important for achieving more efficient high‐throughput scanning.

### File delivery and organization

At some point following a successful scan, images must be delivered to their intended destinations and organized in a meaningful fashion. For many clinical workflows, this may also require integration of the files in the patient record. A key consideration is whether image organization will be conducted at the file system level, within the image management platform, or using a hybrid approach. Organization can occur in a manual fashion, whereby an operator may move a scanned file to centralized location or accession it into an image management system, perhaps also adopting a file naming convention or folder hierarchy reflecting the intended use of the file. Often, much of this may be performed prior to the scan, where filename, user metadata, and destination folders can be applied within the scanner software and attached to scan profiles. By leveraging barcoding, however, many modern scanners can automate these steps. If the slide is barcoded and the barcode contains human‐readable information or a system lookup can be performed to extract necessary information [ideally through an interface with the laboratory information system (LIS)], association with the clinical case has the potential to be automated. Likewise, an opportunity for automated population of metadata content in the image management system can be performed based on barcode lookup in the LIS, for example.

There are often significant challenges to building an interface to clinical systems to perform automatic accessioning or case lookups, so alternatives may be pursued to improve the efficiency of an otherwise manual process. For example, if technicians intend to associate images manually with the case or do not have access to meaningful information from the barcode, automatic file delivery and organization may still improve throughput. If the image is immediately delivered to a central networked repository, multiple staff can have simultaneous access to the image, providing a mechanism for staff that are even working remotely to contribute to the organization of the data; for example, by renaming or organizing files by case number derived from visual inspection of the slide label image without needing physical access to the glass slide. This is still an arduous process but affords the digital pathology laboratory the flexibility to assign the workforce in a dynamic fashion or to outsource some of these duties to personnel who may not be directly involved in the slide scanning operation.

### Quality control (QC)

The quality of an image produced must meet the standard of quality for the intended use case. At minimum, this requires ensuring that the tissue was captured in its entirety and that the image is reasonably free from blur. Although a thorough QC process often considers other factors, such as slide quality (e.g. the presence of air bubbles and excessive slide artifacts such as tissue folds), these should be distinguished from image artifacts that may have been introduced at the time of scanning. For image artifacts, rescanning can often resolve issues without the need for creating a new slide, and therefore belongs to the slide scanning operation rather than the slide generating or data cleaning operations.

To optimize efficiency, the rescan rate should be as low as possible and the rescan trigger should occur before the slide is returned to archives, and ideally occur while the slide is still in the scanner. However, a tradeoff often exists between reducing rescan rate and optimizing scan speed. For example, for scanners that create a focus map prior to scanning, increasing the number of focus points can reduce the prevalence of blurred regions but at the expense of scan time. Likewise, the care that scanning personnel take cleaning and preparing slides can ultimately impact the rescan rate as well, but the amount of time they spend cleaning each slide also dictates how many slides they are able to load per hour. A careful balance between these factors is necessary for optimizing efficiency, and embedding these considerations within a QC paradigm can ensure that the decision does not compromise the quality of the data.

**Figure 3:**
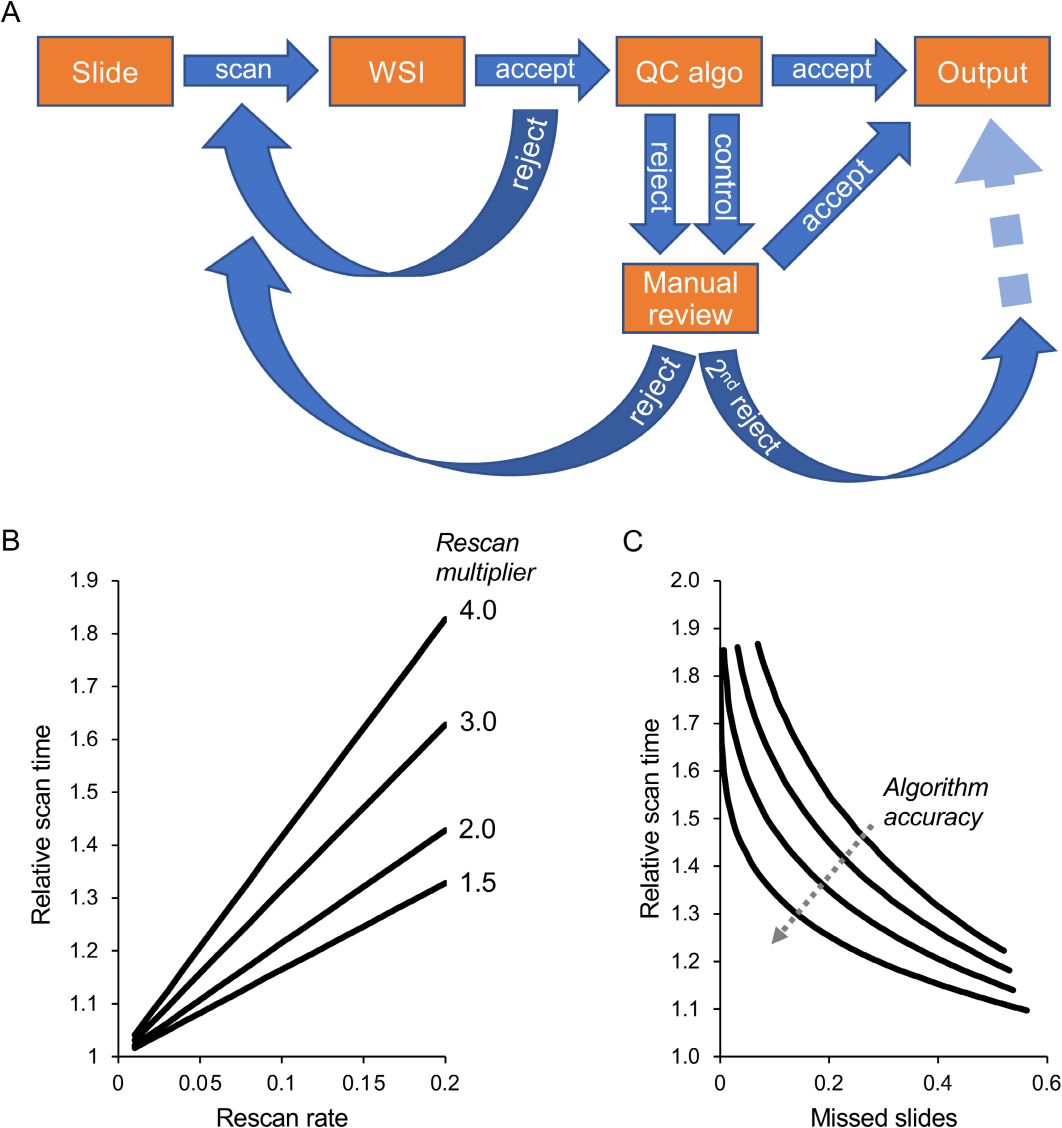
Slide scanning efficiency following AI‐enabled QC strategy. (A) In this AI‐enabled QC strategy, following slide scanning, the WSI is evaluated automatically within the scanner (using the scanner vendor's software) and if rejected is automatically rescanned with a more stringent (but perhaps slower) scan profile. Accepted images pass to a potentially more accurate external QC algorithm which diverts the images to visual review if rejected. The outcome of visual review dictates whether a slide is accepted, rescanned, or rejected without further rescanning for slides in which a rescan has already been attempted. A small number of slides that have passed automated QC are also reviewed visually as a control. (B) The relative scan time of a batch increases linearly as a function of the proportion of slides that undergo auto‐rescan (‘rescan rate’). The model assumed that the more stringent profile took 1.5, 2, 3, or 4 times longer than the original scan profile (‘rescan multiplier’). Simulated scan times were drawn from a Poisson distribution with mean scan time of 60 s, and a constant 10 s slide‐loading time was added to each scan. (C) The sensitivity of the rescan threshold dictates the balance between the scan time of a batch and the proportion of slides that should have been rejected but were missed (‘missed slides’). This balance relies critically on the accuracy of the algorithm to detect such slides, which was modeled by adding Gaussian noise with standard deviation 0.1, 0.15, 0.2, and 0.25 to a simulated quality value uniformly sampled from 0 to 1. A threshold was then applied to the resultant quality value to determine whether a slide will be rescanned. The rescan multiplier was selected to be 2.0 and the rescan rate was 0.05 for this analysis. A thousand simulations were performed for B and C, and mean values are shown.

There are several opportunities after the slide is scanned where quality can be assessed. Figure 3A shows a sample QC workflow utilizing a combination of scanner‐based quality checks, automated image analysis, and visual inspection. In this paradigm, images are initially assessed for quality in an automated fashion by the scanner (for scanners that support this feature); images that fail the quality check are automatically rescanned with a more stringent set of parameters while the slide is still in the scanner. The resultant impact on scan time is modeled and shown in Figure 3B. Alternatively, this automated QC step can be performed externally by independent software, although in this case triggering an automatic rescan can be more difficult or even impossible; instead, the technician may be notified by the automated QC software that the scan quality of a slide failed automated checks and the technician can initiate a rescan under a more stringent profile or on a different scanner.

Slides that pass the initial quality check may then undergo an additional quality check whose purpose is to divert questionable images for manual review. The algorithm used for this purpose may be a scanner‐agnostic algorithm adapted from WSI quality tools such as PathProfiler [14] or HistoQC [15], commercially available tools, or custom in‐house algorithms. The benefit to this second stage is to apply a higher accuracy and more standardized QC protocol, particularly in an environment with multiple slide scanners perhaps from different vendors. Images diverted to manual review using this method then undergo visual inspection and those that are deemed poor quality images will be rescanned, which may also result in further scrutiny of the physical integrity of the glass slide. Importantly, a subset of images that are designated as acceptable by the automated algorithms should be manually reviewed to ensure ongoing reliability of the algorithms; the rescan rate of these should be as close to zero as possible. If a significant number of these control images are found to be lacking in quality, the algorithm or its parameters should be re‐evaluated. Ideally, the control images should be incorporated into the regular visual assessment process in a random fashion to avoid confirmation bias.

The QC method described promotes efficiency by requiring visual inspection of only those images flagged by the algorithm. Care should be taken to establish detection thresholds that ensure that algorithm sensitivity is tuned to meet acceptable standards of the use case; too much sensitivity results in efficiency losses with potentially only marginal improvement in results, whereas low sensitivity can lead to too many problematic images slipping through undetected. The accuracy of the algorithm to detect problematic slides dictates the success of this strategy (Figure 3C). Overall, this procedure shifts the burden of QC to a later stage in the process, which can be performed by personnel who may not be directly involved in the original scan. This can have multiple benefits:

1. Personnel can be specially trained on image quality and QC workflow.
2. QC can be conducted in a scanner‐ or even project‐agnostic fashion.
3. QC can occur at a physically different location than the scan, no longer requiring personnel to be physically present where the material is. This may also be amenable to remote work scenarios, provided regulatory requirements are satisfied.
4. QC can potentially be more reproducible and standardized if the number of QC specialists is less than the number of scanning personnel; for example, if one QC technician reviews all scans. This also decouples bias in QC from bias in slide scanning (e.g. differences in slide preparation procedure between the same technicians who may be reviewing images).
5. Workforce deployment can be more targeted, enabling cross‐trained personnel to dynamically contribute to QC or scanning as priorities shift.

## High‐throughput scanning impacts overall efficiency

For many applications, the slide scanning procedure itself may be one of the main bottlenecks in the digital pathology workflow or data curation process. A shift to high‐throughput whole‐slide scanning creates the opportunity to reduce or even eliminate this bottleneck, which can have ripple effects on other elements of the workflow. For example, in a conventional slide scanning workflow, it may be advantageous for a pathologist or technologist to first preview slides and select representative slides with high importance to be scanned. As many cases can have dozens or even hundreds of slides, this can considerably reduce the overall effort needed in the project. However, in a high‐throughput environment, it is likely that the effort required to scan those slides is less than the effort required for slide review and selection. This has the potential to completely transform the workflow and reduce bottlenecks, especially for archival projects where the primary role of slide selection in the first place may have been to reduce the burden on slide scanning.

Similarly, high‐throughput scanning can enable an overall increase in efficiency that may not have been possible in a conventional scanning workflow. The ability to scan in a high‐throughput manner can reduce scanning turnaround times to a level where it may now be appropriate to integrate slide scanning into a routine pre‐signout workflow, whereas the delay associated with conventional scanning may have previously made this untenable. However, this can be a difficult hurdle to overcome for those who are more accustomed to lower‐throughput scanning and familiar with the challenges of introducing it into a fast‐paced clinical environment.

## Data repository building for AI/ML development

High‐throughput whole‐slide scanning can enable rapid collection of imaging data that when coupled to clinical cases can provide the opportunity to build repositories with clinically relevant metadata. The contribution of high‐throughput scanning can be important for efficiently building repositories at scale, but care should be taken to be mindful of the unique requirements of AI/ML development. For example, AI/ML benefits from diverse data sets that are representative of the intended use case and should capture the slide, tissue, and patient variability likely to be encountered in the real world. Training algorithms on data sets that meet these criteria promotes model generalizability. Despite the care needed to curate data sets for AI/ML development and testing, high‐throughput data collection is key to achieve the sample sizes needed and the diversity of disease entities and patient demographics to create unbiased algorithms. By adopting a high‐throughput slide scanning approach, more resources can be devoted to time‐consuming activities such as identifying cases for curation and annotation.

## Privacy in a high‐throughput environment

Whole‐slide scanning may include the capture of patient information either directly or indirectly to support the clinical implementation of digital pathology and data provenance. For environments using digital slides for patient signout, the College of American Pathologists (CAP) requires that the pathologist confirm that the slide label is correct [16]. Therefore, the slide label image should be connected to the whole‐slide image so that it can be presented alongside the whole‐slide image during the time of viewing and so that verification and validation can be conducted if the images are used after viewing, such as for research. Often, the patient information is located on the slide label and the slide label is usually captured by a pre‐scan macro or label snapshot and not directly imaged as part of the tissue image area. This distinction is important in research applications especially, where only the tissue image area should be made immediately available to investigators. However, it is possible for protected health information (PHI) to encroach upon the tissue image area, depending on slide label placement, and the potential exists for that information to be visible in the whole‐slide image even in transmission microscopy. Therefore, care should be taken to ensure that such images are not released to the investigators without first undergoing a scrubbing process.

Many proprietary whole‐slide image file formats embed the slide label image (and/or macro image, which may include the slide label) within the image file. Image files are moved, copied, and organized as singular units, and the label information necessarily accompanies the tissue image. De‐identification processes can be employed by the image management system at the image viewing level such that the image label is not made accessible. Likewise, application programming interfaces (APIs) can be leveraged in a way that label and macro cannot be exposed. Alternatively, if researchers need direct access to the files or the files are going to be transferred to investigators or to less secure locations, processes should be executed that strip the label and macro images from the image file so that it can be distributed without the accompanying PHI. However, a record of the original label and macro image should remain connected to the tissue image file for the purposes of data provenance. This can be accomplished using a lookup table, hashing mechanisms, or some other method of association.

## Challenges and opportunities

Despite the rapid progress in high‐throughput WSI, significant challenges to achieving optimal efficiency and high quality remain. One of the most important (yet difficult) considerations is how the image is handled after it is scanned. Although modern slide scanners are often designed with the ability to read barcodes, which becomes of great importance to extracting meaningful information about the slide or integrating it into the patient record, not all scanners support barcodes being positioned in non‐label areas. This can lead to a difficult decision for many labs either to forgo reading these barcodes at the time of scanning and adopt manual processes, or to re‐evaluate their barcoding and labeling strategy, which may have other impacts on the lab. As long as barcoding remains such an important tool in practice, more ubiquitous support for barcode reading across the entire slide will be important for streamlining many use cases. A mechanism for identifying labels in non‐standard positions will likewise be important. As noted previously, having the ability to present the slide label to the pathologist is important for viewing, and redacting that slide label from the file is important for de‐identification.

Automated tissue detection and focusing are among the most important examples of automation that have enabled high‐throughput slide scanning. For some slides, particularly those with faintly stained tissue, excessive artifacts, or non‐standard coverslips, these algorithms may consistently fail. This then requires them to be identified in the QC process and rescanned, possibly with manual intervention or another set of parameters that results in longer scan durations. Several innovations in tissue detection and focusing have been described in recent years [17, 18, 19], which may provide an opportunity to improve accuracy if adopted by manufacturers. For example, Bándi *et al* used deep learning to segment tissue from background [17] and achieved a sensitivity of 0.97 when applied to images derived from different tissues and stains. As a demonstration of the impact of technical innovation, they compared the results of this approach with those of more traditional approaches (many of which are used currently by manufacturers, like constant‐ and adaptive‐thresholding) and showed a substantial improvement in accuracy. While this study did not specifically test some of the more difficult slide characteristics that usually impact tissue detection algorithms, such as slide artifacts, faint staining, and adipose tissue, it showed exceptional promise as a generalizable method for reliably capturing tissue in the most common slides. Likewise, a number of different approaches have been published to support autofocusing in WSI [20, 21], often with speed as a key metric. A recent survey of many scanners currently on the market revealed that most use focus map generation as the principal approach [20]; the same review also detailed a number of innovative autofocusing technologies using a combination of optical and/or computational approaches, suggesting an opportunity for potential improvement in this area for commercial devices. Continued innovation by slide scanner vendors to improve automated tissue detection and focusing while being mindful of scan speed is very important for improving efficiency. Likewise, as our model showed, additional improvements in post‐scanning QC algorithm performance will also go a long way toward enhancing efficiency.

Finally, one of the fundamental requirements of an efficient workflow is to allow technicians to load slides into the scanner and trust that they will be scanned so that they can prepare the next batch of slides to be scanned, or can QC images that have already been scanned, or can work on other things. Similarly, some technicians will load a batch of slides before the end of their shift, expecting that they will be complete when they return the next day. This is derailed when the slide scanner halts due to a problem, often due to a mechanical fault (but not always). Improving the robustness of slide scanning and its resilience to issues that give rise to catastrophic failures that halt the entire operation should be of high priority to all vendors.

## Conclusions

WSI presents an exciting opportunity to deploy digital clinical workflows, histologic quantification, and conduct AI/ML model development but can be a time‐consuming and costly endeavor at large scales. Recent advances in WSI, heralded by higher capacity scanners, shorter scan times, and more automation, have enabled large data curation efforts and have enabled large centers to digitize their slides for the purposes of primary or remote signout, archival, or routine quantitative tools. Continued improvements in WSI technology will further improve efficiency, likely driving WSI adoption and making large‐scale data curation more tractable.

## Author contributions statement

MDZ and KRA prepared the manuscript and figures.
